# A content analysis of Canadian influencer crisis messages on Instagram and the public’s response during COVID-19

**DOI:** 10.1186/s12889-022-13129-5

**Published:** 2022-04-15

**Authors:** Melissa MacKay, Caitlin Ford, Taylor Colangeli, Daniel Gillis, Jennifer E. McWhirter, Andrew Papadopoulos

**Affiliations:** 1 Department of Population Medicine, University of Guelph, Guelph, ON N1G2W1 Canada; 2 School of Computer Science, University of Guelph, Guelph, ON N1G2W1 Canada

**Keywords:** Crisis communication, COVID-19, Social media, Health Belief Model, Extended Parallel Processing Model, Engagement analysis, Sentiment analysis

## Abstract

Successful mitigation of emerging infectious disease requires that the public adopt recommended behaviours, which is directly influenced by effective crisis communication. Social media has become an important communication channel during COVID-19 where official actors, influencers, and the public are co-creating crisis messages. Our research examined COVID-19-related crisis messages across Canadian influencer accounts within news media, politicians, public health and government, science communicators, and brand influencer and celebrities, posted on Instagram between December 2019 and March 2021 for Health Belief Model and Extended Parallel Processing Model constructs and the corresponding public comment sentiment and engagement. Thirty-three influencer accounts resulted in a total of 2,642 Instagram posts collected, along with 461,436 comments, which showed overall low use of constructs in both captions and images. Further, most posts used no combinations (*n* = 0 or 1 construct per post) of constructs in captions and images and very infrequently used captions that combined threat (severity and susceptibility) with cues to action and efficacy. Brand influencers and celebrities, politicians, and science communicators had above average post engagement while public health and government and news media had lower. Finally, most influencers saw the largest proportion of neutral sentiment comments. Crisis messages must be designed to include combinations of constructs that increase message acceptance and influence risk perception and efficacy to increase the adoption of recommended and mandated behaviours.

## Background

### Crisis communication

Behaviour change is an important outcome of many public health initiatives, including those surrounding the COVID-19 pandemic. Successful mitigation of COVID-19 requires that the public follows public health recommendations, including mask wearing, physical distancing, and getting vaccinated. Effective crisis communication can increase adherence to public health measures, which is necessary to reduce the burden of COVID-19 and other public health emergencies. Effective crisis communication is an essential element of the strategic response to COVID-19 where people are empowered to follow recommendations. Not only is effective crisis communication essential for the shorter term uptake of public health recommendations, but it has important longer term impacts including preventing pandemic fatigue, encouraging vaccine uptake and engagement with the healthcare system, and importantly, maintaining trust [1].

Practice and research have resulted in guiding principles for effective crisis communication that should be used to increase acceptance of and adherence to messages. Guiding principles for effective communication include communicating uncertainty and with transparency, being first and empathic, and ensuring information is clear and targeted to various subpopulations [2–5]. However, message acceptance and the adoption of public health recommendations is moderated by social, cultural, and behavioural factors during a crisis [6, 7]. Risk and threat perception, as well as the social and individual contexts play a role in how information is processed and ultimately acted upon during a crisis [6]. Crisis messages must be evaluated for inclusion of guiding principles and theory-based messaging regarding threat perception and behaviour change, as well as the public’s reaction to assess communication effectiveness [8]. Publicly available crisis communication messages on social media provide an ideal opportunity to assess the public’s acceptance of crisis messages through comments and engagement [8].

### Crisis communication on social media

In Canada, governments, public health and healthcare, and other actors at all levels have implemented measures to slow the spread of COVID-19. Issues with distrust among the public [9, 10], criticisms regarding lack of transparency [11], and inconsistent and unclear communications [12, 13], as well as the current infodemic [14, 15] have undermined the efforts of official actors. An infodemic is described as an overabundance of information, including false or misleading information during disease outbreaks [16]. Communication efforts included warnings about the nature and severity of COVID-19, how to prevent infection, and information about mandatory measures like stay-at-home orders across the provinces. Communication efforts were not limited to official actors, as social media enables individuals to share official messages as well as create their own regarding COVID-19.

Social media is an important communication channel for crisis communication, especially during COVID-19, and Canadians are spending more time on social media than ever before [17]. A 2019 Canadian emergency preparedness indicators report included social media as an essential aspect of communication strategy to provide information, monitor the infodemic, and engage with the public [18]. Social media platforms allow for access to an unprecedented amount of content and can have a large influence on behaviour [19]. In 2020, Instagram was the fourth most popular social media platform in Canada with 51% of Canadians having an account and 69% of those accessing the site daily [20]. Young people [18–24] remain the largest adopters of social media and 18–24 and 25–34 year olds are the dominant groups on Instagram [20]. By Fall 2021, people under the age of 19, followed by 20–29, and then 20–39 year olds made up the largest proportion of COVID-19 cases in Canada [21]. Coupled with the fact transmission of newer variants during the third and fourth waves in Canada is occurring more in children and youth [22], the crisis communication on this channel that is widely used by an important subpopulation should be evaluated.

Social media provides an excellent channel through which to share information quickly to subpopulations by targeting and tailoring information and selecting appropriate platforms [23]. Partnerships between official actors, such as public health, with those that can better reach subpopulations, such as brand influencers and celebrities, can help amplify official messages. Influencers are people who exert influence, guide the actions of others, and are able to generate interest in something [24]. The Social Mediated Crisis Communication Model holds that many publics exist within a crisis and identifying those that have a large number of followers with high engagement should be leveraged to increase amplification of messages and impact risk perception [25]. Brand influencers have dedicated and engaged followers and collaborate with brands on social media to promote a product or service [26, 27]. They have been used much less in partnership with public health but should be considered as they play a role in influencing health-related behaviours [26]. Influencers on social media are also creating their own messaging and influence with regards to emerging infectious disease and should be taken into account [28].

Social media monitoring provides important evidence about the impact of social media communication. Analyzing data gives insight into how content is preforming with audiences, identifying key influencers, analyzing trends, and identifying what strategies work best for different audiences [29]. Many of the metrics are available for social media account managers to explore, while some basic metrics such as followers, sentiment, engagement, activity, and amplification are available publicly. Sentiment analysis is a process used to determine the emotional tone behind a series of words and emoticons [30]. The analysis of comments posted by the public to crisis messages provide important information about the public’s acceptance of messages and uptake of recommended behaviours [8]. Further, engagement metrics signal interaction with the content and can be used by the public to evaluate the information [31].

### Behaviour change models to guide messaging

Given the complexity of crisis communication and its ultimate goal of ensuring the adoption of public health measures, theory-driven public health messaging may be more effective to persuade individuals to follow recommended behaviours [32]. One of the most widely used models to explain health behaviour is the Health Belief Model (HBM) [32–34]. The HBM theorizes people will adopt a behaviour to prevent disease if they think they are susceptible, they believe the disease would be severe, they believe there are positive benefits to taking action that are greater than the barriers to action, and finally they have confidence to succeed in taking action (self-efficacy) after exposure to factors that prompt action (cues to action) [32–34]. Each of the six constructs provide opportunities to improve crisis communication to influence risk perception and motivate individuals to adhere to risk protective measures [32].

Additionally, the Extended Parallel Processing Model (EPPM) conceptualizes aspects of the HBM to explain how message components including perceived severity and susceptibility, and efficacy can lead to either rejection or adoption of protective behaviours [35–37]. Two processes result from combinations of high and low threat, fear, and efficacy: 1) danger control process where the message is accepted and protective action is taken when high threat and high efficacy exists; or 2) fear control where the message and protective action is rejected when low threat and low efficacy exists [35, 36]. When the perceived threat is high, which is influenced by severity and susceptibility information, but efficacy is low, fear results in a defensive response and the rejection of the message [36]. Maladaptive responses, such as rejection of messages and not taking action, result from inadequate information about the threat resulting in fear, combined with inadequate information about efficacy resulting in the ability to take action [38].

The HBM and EPPM have been used to assess social media messages during emerging infectious diseases by examining the presence of the constructs of each model and the relationship with message transmission, effectiveness, and behaviour intentions. For example, a quantitative content analysis of vaccine-related influenza tweets was analyzed for HBM constructs and user engagement during the 2018 and 2019 flu seasons and found messages contained high fear content but low efficacy content and resulted in low engagement [39]. A COVID-19-related study assessed Twitter messages posted by public agencies for HBM constructs and retransmission metrics and found messages about severity and susceptibility positively impact retransmission [40]. Another recent COVID-19 study assessed the relationship between social media exposure and risk perception through the lens of the EPPM and found that both risk and efficacy together in crisis messages leads to preventative behaviours [41]. Finally, a study that examined the use of susceptibility, severity, and response efficacy information related to COVID-19 on TikTok videos shared by accounts of 8 public health and United Nation agencies found that videos that included susceptibility, severity, and response efficacy information had higher engagement than those that did not [42].

### Current research

Much of the research using the HBM and EPPM has been to evaluate whether the constructs are effective at influencing behaviour change, usually by involving participants in the research to assess how they perceive constructs in messages and their corresponding behavioural intentions [36]. However, less research has centered on evaluating whether official crisis communication includes important constructs from the HBM and EPPM, especially on social media. One study evaluated 1000 tweets from the public regarding the flu vaccine and inclusion of HBM constructs (Guidry et al., 2020), while another examined 1409 twitter messages sent by public health authorities regarding Zika for EPPM constructs [43], while another used machine learning to look at HBM constructs used in Facebook posts regarding COVID-19 by public health authorities [44]. No research has focused on Canadian actors and influencers, herein referred to as influencers, and their use of HBM and EPPM constructs with social media crisis communication and how the public has responded to these messages.

The aim of this research is to describe and compare how different Canadian influencers on Instagram are incorporating HBM and EPPM constructs in their COVID-19-related crisis messages and how the messages are being received by publics.

The objectives of this research include:

1. To describe the number of COVID-19 Instagram posts, average number of comments, average number of loves, average number of replies, and average post engagement rate across influencer categories (i.e., news media, politicians, public health and government, science communicators, and brand influencers).
2. To describe the trinary sentiment (i.e., positive, neutral, negative) of comments related to included Instagram posts by influencer category.
3. To describe the use of severity, susceptibility, benefits, barriers, and cues to action/efficacy in COVID-19 Instagram text captions across influencer categories.
4. To describe the use of severity, susceptibility, benefits, barriers, and cues to action/efficacy in COVID-19 Instagram images across influencer categories.
5. To describe the presence of the EPPM danger control and fear control processes in COVID-19 Instagram captions and images across influencer categories.

## Methods

### Ethics

As per the University of Guelph’s Research Ethics Board, ethics approval for this study was not required as it used publicly available Instagram pages.

### Inclusion criteria

Posts made between December 31, 2019 (i.e., first case of pneumonia without a known case identified) and March 3, 2021 (i.e., day prior to data collection) were included when the post was in English, was an Instagram post (i.e., not a reel, highlight, or story), and related to COVID-19 (i.e., the post or image either directly mentions COVID-19 or depicts a public health measure related to COVID-19 such as mask wearing, physical distancing, thanking a front-line worker, supporting the local economy, etc.). Additionally, influencers residing or operating in Canada that represented an actual person or organizational account were included when they were macro-level influencers with no less than 100,000 followers or were relevant to the federal level. Posts were excluded if the comments were turned off the post, posts that contained videos rather than a picture, or advertisement posts.

### Data collection

Instagram posts related to COVID-19 from influencers across five categories (news media, politicians, public health and government, science communicators, and brand influencers and celebrities) were manually collected by three researchers in March 2021. Influencers relevant to the federal level were chosen for news media, politicians, and public health and government. Science communicators and brand influencers and celebrities were chosen using HypeAuditor (HyperAuditor, 2021) and StarNGage, which is no longer available at the time of writing. Influencer ranking works but analyzing real followers and authentic engagement (likes and comments that come from real people rather than bots) daily for accounts with more than 10,000 followers to compare influencers with the highest following and engagement [45]. The top twenty influencer accounts of all categories and top ten influencer accounts of ‘health and medicine’ and ‘health and fitness’ subcategories were explored to examine relevance to study inclusion criteria. The list of included accounts organized by influencer category can be found in Table 1.

**Table 1:** Influencer accounts included by influencer category

News Media	Politicians	Public Health and Government	Science Communicators	Brand Influencers and Celebrities
@ctvnews	@justinpjtrudeau	@healthycdns	@oncovid19	@vancityreynolds
@cbcnews	@erintoolemp	@cihr_irsc	@scienceupfirst	@erintoolemp
@globalnews	@pattyhajdu	@statcan_eng	@sajjadfazel	@thebirdspapaya
@globeandmail	@jagmeetsingh	@gacanada.amcanada	@science.sam	@celinedion
@nationalpost	@annamiepaul		@caulfieldtim	@jillian.harris
@huffpostcanada			@asapscience	@instadanjlevy
			@yournursingeducator	@sierrafurtado
			@thegirlymd	@claudiatihan
				@albeaton

Each included influencer’s Instagram page was accessed, and posts related to study inclusion criteria were manually collected including the account information (number of followers, biography, and category of influencer), total number of posts during inclusion dates, post caption, post image captured by screenshot, number of comments, number of loves, and comments and replies on included posts. Comments and replies were automatically collected using the Phantombuster Instagram Post Commentors automation, which accesses the Instagram Application Programming Interface (Phantombuster, 2021).

An identification letter was created for each influencer and a combination of the identification letter and a unique number was used for each post. An Excel spreadsheet [46] was used to collect the post caption and included engagement information.

### Post engagement

The number of comments, loves, and replies were collected, as well as total number of followers for each influencer.

A post engagement rate was calculated for each post by totalling engagements by post (number of loves + number of comments) dividing by total number of followers, multiplied by 100 [47]. The post engagement rate measures the amount of interaction each post receives relative to the influencer’s following [47]. An average post engagement rate was calculated for each influencer and then across each influencer category.

### Sentiment analysis

SentiStrength (Java version) was used to conduct a trinary sentiment analysis of follower comments [48]. Each comment was defined as positive, neutral, or negative by assigning each word in a short string of text a numerical sentiment score on a scale of positive (+ 1 not positive to + 5 extremely positive) to negative (-1 not negative to -5 extremely negative) [49]. A word’s sentiment score of + 1 or -1 indicates neutral sentiment. To assign an overall trinary sentiment, the program determines the difference between the most positive and most negative word in the text [49].

To improve the accuracy of the results, some of the word’s pre-assigned scores were modified as they were inaccurately driving negative results, which can occur during highly specific events such as the COVID-19 pandemic [50]. For example, before altering the sentiment of some word’s pre-assigned scores, a comment that read “The COVID-19 vaccine protects people from dying” is assigned a negative overall sentiment. This is because ‘dying’ is assigned a negative score of -2 in the programs pre-assigned scores even though in the example it is not meant in a negative way. The following words were changed from negative (-2 to -5) to neutral (-1): death, dying, emergency, ill, infect, isolate, risk, sick, disease, illness, combat, headache, fever, symptom, and dead. These words are commonly used regarding COVID-19, but not always in a negative context. The program’s acronym lists, idiom list, spelling correction list, booster word list, negating word list, emoticon list, and standard settings were used.

### Content analysis

The constructs of the HBM and EPPM were used to assess crisis communication messages for constructs of behaviour change and risk perception models that can predict message acceptance and adoption of behaviour change. The constructs and corresponding definitions can be found in Table 2. As the evaluation of crisis messages for HBM and EPPM constructs do not include assessing individual perceptions, the perceived aspect of constructs is removed. Constructs are operationalized to be able to examine messages for important aspects of each construct. Cues to action and efficacy have been combined to capture elements from both the HBM (self-efficacy and cues to action) and EPPM (self-efficacy and response efficacy) so that messaging about prompts or steps an individual can take or the effectiveness of public health measures are captured. Codes were distinct but not mutually exclusive, meaning a caption or post could be coded for one or all constructs and other variables.

**Table 2:** Health belief model constructs, definitions, and example captions

Construct	Definition	Example Caption
Severity	Posts that indicated an assessment of an increase in the perceived seriousness and consequences of contracting coronavirus disease (eg, hospitalization, pneumonia, death, mortality risk, variants)	U.K Prime Minister Boris Johnson was moved into the intensive care unit of a London hospital after his COVID-19 symptoms worsened Monday, just a day after he was admitted for what were said to be routine tests
Susceptibility	Posts that indicated an assessment of the increased likelihood of contracting coronavirus disease, highlighting increasing local prevalence and the high number of imported cases	This is Violet Violet lost her best friend- her “Gramma”- Linda Lee Gruntke in 2018 Violet is worried about COVID’s impact on seniors She doesn’t want to lose any more Gramma’s
Benefits	Posts that supported public health measures (eg, school closure, working from home, cancellation of events and mass gatherings, vaccination) to reduce the transmission of coronavirus disease	To get through this together, we must all stay apart. #PhysicalDistancing means you can still connect with loved ones, but do it virtually. #StayHOmeSaveLives. #COVID19 #FlattenTheCurve
Barriers	Posts that mentioned the difficulties, challenges, and negative effects of adhering to public health measures (eg, loss of freedom, violation of individual rights, inconvenience, loss of income)	Happy Friday from the South African rain frog. Send this to someone you are missing whist STAYING HOME I QUAR QUAR!
Cues to action and response efficacy	Posts that include cues to action or prompts to engage in a behaviour (reminder to self-screen, get a covid-19 test if you have symptoms, talk to Dr., get the vaccine, register for the vaccine). Posts that include messages around efficacy or messages about how effective the proposed action is	If you’re feeling sick, protect yourself and others by staying home. If you need to travel, make sure you check out the travel advisories for your planned destination before you leave! Especially given the current #coronavirus situation

Adapted from Raamkumar, Tan, & Wee, 2020

A codebook describing each construct was created and a codebook training session with the involved researchers occurred before coding began. Two researchers independently coded a 10% random sample of the data (*n* = 265) and captions (*n* = 256) separately for the HBM constructs using NVivo 12 Plus [51]. Pre-testing for coding was completed until a kappa of > 0.8 was achieved for inter-coder reliability, and all conflicts were discussed and resolved before the remaining data was split equally among the researchers and coding was completed.

### Statistical analysis

Data were collated so each post was labelled according to its influencer category, number of instances of HBM constructs used in post captions and images, as well as the number of comments on each post labelled as having positive, neutral, and negative sentiment. Data were aggregated and evaluated using chi-square tests to identify differences across sources, HBM constructs used in captions and images, and sentiment. Data were analyzed in SPSS 26 [52].

## Results

A total of 2,642 COVID-19-related Instagram posts were collected based on the inclusion criteria across 32 influencer accounts. A total of 461,436 comments and replies related to the included posts were collected.

### Post engagement

Across all influencer categories, brand influencers and celebrities had the most followers and the highest number of loves (Table 3). Public health had the least number of followers and posted the largest percent of COVID-19-related posts but had the lowest number of average comments. Politicians had the second highest number of followers and the largest average number of comments. News media had the second highest average number of comments and replies.

**Table 3:** Engagement across influencer categories

Influencer Category	Total Followers	Average COVID Posts/Total (%)	Average Number of Comments	Average Number of Loves	Average Number of Replies	Average Post Engagement Rate (%)
News Media	1,606,400	32.04	15,743	423,220	34	0.64
Politicians	4,581,800	29.10	46,719	1,779,969	14	2.94
Public Health and Government	185,452	55.47	1,523	29,986	13	0.78
Science Communicators	1,204,787	39	1,680	94,542	161	2.87
Brand Influencers and Celebrities	52,584,000	4.11	12,633	5,680,071	19	5.04

In terms of the average post engagement rate, brand influencers and celebrities had the highest post engagement rate (5.04), followed by politicians (2.94), science communicators (2.87), public health and government (0.78), and finally news media (0.64). See Table 2 for engagement rate across influencer categories.

The pattern of sentiment differed across influencer types (Table 4) and was statistically significant.

**Table 4:** Trinary sentiment of comments related to COVID-19 instagram posts by influencer category

	Sentiment		
Influencer Category	Positive n(%)	Neutral n(%)	Negative n(%)
News Media	22,367 (34%)	27,902 (43%)	14,960 (23%)
Politicians	51,474 (31%)	84,087 (51%)	28,965 (18%)
Public Health and Government	878 (31%)	1,210 (43%)	715 (26%)
Science Communicators	5,246 (49%)	3,712 (34%)	1,831 (17%)
Brand Influencers and Celebrities	31,762 (40%)	40,790 (52%)	6,648 (8%)

*X*^2^ = 21.387 on 8 degrees of freedom, *p* < 0.05

(*p* < 0.05). The trinary sentiment analysis of comments and replies made on included Instagram posts showed the largest percent of comments were neutral for news media (43%), politicians (51%), public health and government (43%), and brand influencers and celebrities (52%). Science communicators was the only influencer category that saw the largest percentage of comments classified as positive (49%). The percentage of negative comments compared to positive and neutral was lowest across all influencer sources.

### Content analysis

#### Health belief model constructs in captions and images

The use of HBM constructs across influencer categories varied across post captions and images. In post captions (Table 5), severity was used least in posts by politicians (6%), public health and government (2%), science communicators (6%), and brand influencers and celebrities (2%). Cues to action/efficacy was used least in posts made by news media (9%). News media, politicians, and brand influencers and celebrities used barriers the most in their post captions (34%; 34%; 41% respectively). Cues to action were included most frequently in post captions by public health and government (33%) and science communicators (37%). Comparing the relationships between the use of HBM constructs in captions and influencer types was found to be statistically significant.

**Table 5:** Use of health belief model constructs across influencer categories in instagram post captions

Influencer Category	Severity n (%)^1^	Susceptibility n (%)^1^	Benefits n (%)^1^	Barriers n (%)^1^	Cues to Action/Efficacy n (%)^1^	Total Constructs Used
News Media	179 (15%)	208 (17%)	301 (25%)	414 (34%)	103 (9%)	1,205
Politicians	20 (6%)	42 (13%)	71 (21%)	113 (34%)	88 (26%)	334
Public Health and Government	9 (2%)	27 (6%)	147 (30%)	144 (30%)	159 (33%)	486
Science Communicators	31 (6%)	62 (13%)	97 (20%)	121 (25%)	182 (4%)	493
Brand Influencers and Celebrities	1 (2%)	2 (3%)	20 (31%)	26 (41%)	15 (23%)	64

^1^% determined by dividing the use of each construct by the total number of uses of constructs across influencer category

*X*^2^ = 334.166 on 16 degrees of freedom, *p* < 0.05

In post images (Table 6), severity was used least in posts made by politicians (9%), public health and government (2%), science communicators (7%), and brand influencers and celebrities (0%). Cues to action/efficacy was used least by news media (6%). News media used benefits (35%), politicians, science communicators, and brand influencers and celebrities used cues to action and efficacy (39%; 41%; 50% respectively), public health and government used barriers (39%) most frequently in post images. The relationship between HBM constructs in post images and influencer category was found to be statistically significant.

**Table 6:** Use of health belief model constructs across influencer categories in instagram post images

Influencer Category	Severity n(%)^1^	Susceptibility n(%)^1^	Benefits n(%)^1^	Barriers n(%)^1^	Cues to Action/Efficacy n(%)^1^	Total Constructs Used
News Media	85 (19%)	56 (13%)	155 (35%)	127 (28%)	25 (6%)	448
Politicians	9 (7%)	14 (11%)	14 (11%)	23 (18%)	39 (30%)	99
Public Health and Government	4 (5%)	12 (14%)	39 (46%)	65 (77%)	47 (28%)	167
Science Communicators	27 (8%)	58 (16%)	56 (16%)	71 (20%)	149 (41%)	361
Brand Influencers and Celebrities	0 (0%)	1 (7%)	4 (29%)	2 (14%)	7 (50%)	14

^1^% determined by dividing the use of each construct by the total number of uses of constructs across influencer category

*X*^2^ = 221.794 on 16 degrees of freedom,* p* < 0.05

#### Combination of HBM constructs used in captions and images

When examining the number of HBM constructs used per post caption (Table 7), posts with no HBM constructs (*n* = 0) and thus no combinations of constructs, were the most common for all influencer categories except public health, government and news media for which one was the most common number of HBM constructs in post captions. Influencer categories made relatively few posts with HBM construct combinations (*n* = 2 or more constructs). Post captions with 5 constructs were between 0 posts (politicians, public health and government, and brand influencers and celebrities) to 1 post (news media and science communicators).

**Table 7:** Use of health belief model constructs in combination in captions

Number of Health Belief Model Constructs Per Post	News Media *n*(%)	Politicians *n*(%)	Public Health and Government *n*(%)	Science Communicators *n*(%)	Brand Influencers and Celebrities *n*(%)	Average *n*(%)
0	320 (30%)	248 (52%)	133 (30%)	270 (46%)	40 (46%)	1011 (38%)
1	364 (35%)	139 (29%)	163 (37%)	196 (33%)	35 (40%)	897 (34%)
2	270 (26%)	67 (14%)	109 (25%)	91 (15%)	8 (9%)	545 (21%)
3	86 (8%)	15 (3%)	31 (7%)	30 (5%)	3 (4%)	165 (6%)
4	9 (0.9%)	4 (1%)	3 (1%)	5 (0.8%)	1 (1%)	22 (0.8%)
5	1 (0.1%)	0 (0%)	0 (0%)	1 (0.2%)	0 (0%)	2 (0.01%)

Similarly, when examining the number of constructs used per post image (Table 8), most frequently images contained no HBM constructs, which the case across influencer categories. As with captions, relatively few posts had HBM construct combinations (*n* = 2 or more constructs). With only one exception, no images contained 4 or 5 HBM constructs across influencer categories.

**Table 8:** Use of health belief model constructs in combination in images

Number of Health Belief Model Constructs Per Post	News Media *n*(%)	Politicians *n*(%)	Public Health and Government *n*(%)	Science Communicators *n*(%)	Brand Influencers and Celebrities *n*(%)	Average *n*(%)
0	675 (64%)	392 (83%)	296 (67%)	315 (53%)	75 (86%)	1753 (66%)
1	318 (30%)	64 (14%)	120 (27%)	208 (35%)	10 (12%)	720 (27%)
2	49 (5%)	16 (3%)	22 (5%)	57 (10%)	2 (2%)	146 (6%)
3	7 (0.7%)	1 (0.2%)	1 (0.2%)	13 (2%)	0 (0%)	22 (0.8%)
4	0 (0%)	0 (0%)	0 (0%)	0 (0%)	0 (0%)	0 (0%)
5	1 (0.1%)	0 (0%)	0 (0%)	0 (0%)	0 (0%)	1 (0.04%)

#### Presence of threat and efficacy/cues to action in captions and images

Threat (susceptibility and severity) and efficacy (efficacy and cues to action) were examined in combination (Table 9) in captions to assess the danger control process of the EPPM which risk messages should initiate. All categories of influencers very infrequently shared captions that combined threat and efficacy. The highest frequency was found for the combination of susceptibility and cues to action/efficacy by news media (*n* = 38 or 3.6% of total constructs for news media), followed by science communicators for severity and cues to action/efficacy (*n* = 13 or 2.2% of total constructs for science communicators). The combined presence of severity and susceptibility and cues to action/efficacy was highest among news media (1.2%), followed by science communicators (0.8%), and public health and government (0.7%). A statistically significant (*p* < 0.05) relationship was found between the combinations of threat and efficacy messages and source.

**Table 9:** Use of threat and efficacy combined in instagram post captions

Threat and Efficacy Constructs	News Media *n* (%)^1^	Politicians *n* (%)^1^	Public Health and Government *n* (%)^1^	Science Communicators *n* (%)^1^	Brand Influencers and Celebrities *n* (%)^1^
Severity and Cues to Action/Efficacy	22 (2%)	5 (1%)	4 (0.9%)	13 (2.2%)	1 (1.1%)
Susceptibility and Cues to Action/Efficacy	38 (3.6%)	0 (0%)	0 (0%)	8 (1.3%)	0 (0%)
Severity and Susceptibility and Cues to Action/Efficacy	13 (1.2%)	2 (0.4%)	3 (0.7%)	5 (0.8%)	0 (0%)

^1^ Percentage of post captions using both constructs indicated

*X*^2^ = 17.202 on 8 degrees of freedom, *p* < 0.05

Threat and efficacy were examined in combination (Table 10) to assess the influence on the danger control process of the EPPM within post images as well. As seen in the captions, influencers very infrequently shared images that had the presence of both threat and efficacy. The highest frequency was found for susceptibility and cues to action/efficacy combined, shared by science communicators (*n* = 14 or 2%). Only news media (*n* = 2 or 0.2%) and science communicators (*n* = 3 or 0.5%) shared images that combined both threat and cues to action/efficacy. The relationship between constructs and source was not found to be statistically significant.

**Table 10:** Use of threat and efficacy combined in instagram post images

Threat and Efficacy Constructs	News Media *n* (%)^1^	Politicians *n* (%)^1^	Public Health and Government *n* (%)^1^	Science Communicators *n* (%)^1^	Brand Influencers and Celebrities *n* (%)^1^
Severity and Cues to Action/Efficacy	5 (0.5%)	0 (0%)	0 (0%)	8 (1%)	0 (0%)
Susceptibility and Cues to Action/Efficacy	3 (0.3%)	4 (0.8%)	5 (1%)	14 (2%)	0 (0%)
Severity and Susceptibility and Cues to Action/Efficacy	2 (0.2%)	0 (0%)	0 (0%)	3 (0.5%)	0 (0%)

^1^ Percentage of post images using both constructs indicated

*X*^2^ = 9.836 on 6 degrees of freedom, *p* = 0.13

## Discussion

This research analyzed COVID-19-related crisis communication on Instagram by Canadian influencers including news media, politicians, public health and government, science communicators, and brand influencers and celebrities. Our analysis examined whether influencer crisis communication employed HBM and EPPM constructs within Instagram captions and images, as well as the corresponding engagement rate and sentiment of public comments in response to the posts. Our research found that across influencers, the HBM constructs are not being widely incorporated into captions and images, especially in combination. Further, the combination of threat appeals and efficacy, which elicit the danger control process, are rarely incorporated in Instagram captions across influencer categories. Finally, in terms of public response to messaging, neutral sentiment of comments to COVID-19-related Instagram posts was the most common for all influencer types except for science communicators, for whom positive sentiment comments were the most common. Average post engagement rate was highest for brand influencers and celebrities and lowest for news media and public health and government.

Much of the prior research examining the use of HBM constructs in crisis communication is experimental and focused on examining social media posts made by publics for the constructs. For example, Meadows et al. (2019) examined 3000 tweets posted by publics during the California measles outbreak for HBM constructs. They found individuals were more likely to discuss severity, while organizations were most likely to offer cues to action [53]. Other studies conduct surveys to assess how HBM constructs influence behaviour, such as Ranjit et al. (2021) cross section survey of U.S. adults during COVID-19 which found cues to action influenced staying at home while severity and susceptibility influenced social distancing [54]. While this research is important for understanding how perception of the constructs influences behaviour, it is also important to understand how the public reacts to crisis communication messages [8], including those that contain HBM and EPPM constructs. Our examines the public’s reaction to crisis messages posted by influencers on Instagram through comment sentiment and engagement, rather than through examining social media posts made by the public during crises.

### Response to COVID-19-related messaging is concerning

In terms of post engagement rate, the average rate per post across all types of posts and accounts was 2.02% in 2020 [55]. Interestingly, engagement rates vary with industry as higher education organizations are typically higher (average of 3.56%) and brand influencers and celebrities are lower (1.67%) [56] but higher follower count makes it more difficult to achieve higher engagement rates [47]. Our research found that brand influencers and celebrities, science communicators, and politicians had above average post engagement rates. News media and public health and government had lower than average post engagement rates, meaning followers were not as engaged with their content. Similarly, a study of various levels of Canadian government and public health during COVID-19 found that the Prime Minister was by far the most engaged with on Twitter and Facebook and federal public health saw much lower levels of engagement [57]. Teichmann et al. (2020) also found that celebrities and brand influencers that share public health messages saw high levels of engagement.

Another COVID-19-related study found that celebrities who shared their lived experience influenced risk perception and reinforced public health recommendations [58]. A study examining influenza vaccine uptake found that partnering with social media influencers had a positive impact on changing perceptions and uptake of the flu vaccine [59]. Increasing engagement is important as the Instagram algorithm will promote the content within followers feeds, and importantly for public health, higher engagement means more credibility and trust with the public [60]. Partnering with influencers like celebrities, brand influencers, and science communicators who have large followings and strong connections with their followers is important to amplify accurate public health information, influence risk perceptions, reach subpopulations, and increase credibility and trust [58, 59, 61, 62]. The current study found high engagement and very low negative comments on the COVID-19-related posts for brand influencers but low HBM and EPPM constructs. Additionally, we found high positive sentiment and higher than average engagement on COVID-19-related posts by science communicators. While it is not surprising that brand influencers would not focus on incorporating public health models and theories, brand influencers and science communicators provide an important potential partner and trusted spokesperson during crises. Partnerships between public health, government, and brand influencers and science communicators allow public health to provide accurate messages that reflect risk perception and influence behaviour change to influencers with larger followings and strong audience engagement.

Monitoring social media for sentiments and emotions is another important way for actors to assess the effectiveness of their crisis communication [63]. Our research found the largest proportion of comments were classified as neutral for all influencer categories except for science communicators, for which the largest proportion were classified as positive. Comments classified as negative made up approximately one-quarter for news media, politicians, and public health and government comments. While negative comments were proportionally lower for all influencer types, negative comments have a stronger effect on perception of the information and source compared to positive [64]. An analysis of Facebook pages of public health organizations during COVID-19 in Singapore, the United States, and England found negative comments were the most prevalent [63]. Another analysis of Canadian public health and news media Facebook pages found negative comments also made up the largest number across the different sources [4]. The sentiment of comments to crisis communications is important to assess as prior studies have found readers use other comments to assess source credibility [65]. Further, Winter et al. (2015) found that negative comments made on news media Facebook posts had persuasive effects on the views of others. Importantly for crisis communication, the exemplification effect of negative comments can influence the public perception of risk, as well as the credibility of actors [64].

### HBM and EPPM constructs were not widely or consistently found in instagram captions or images

Overall, the most frequent use of a construct was barrier-related information on Instagram captions made by news media (35%), politicians (34%) and brand influencers and celebrities (41%). Susceptibility information was not widely included with 17% or less of captions and 16% or less of images across influencer categories. Benefits, barriers, and cues to action/efficacy were incorporated inconsistently between influencer categories and captions and images. Past research examining influenza vaccine behaviour found that severity, susceptibility, barriers, and cues to action/efficacy together were significantly related to intention to get vaccinated (Guidry et al., 2020). Another study that examined COVID-19 vaccine behaviour found that self-efficacy was an important predictor of vaccine behaviours and play a mediating role with other constructs including barriers, benefits, and cues to action [33]. Cues to action/efficacy was used in between 9–37% of captions and 6–50% of images, although 50% of images translates to seven instances of this construct in brand influencer images. During the early stages of the COVID-19 pandemic, a study on Twitter messages posted by 690 accounts representing public health, emergency management, and elected officials found that messages containing severity and susceptibility information as well as cues to action and efficacy strongly influenced message retransmission [40]. Not only are the constructs important for influencing risk perception, message acceptance, and public health measure uptake, but they also influence how much individuals will share the messages within their social networks.

Influencer categories saw most posts with no combination (*n* = 0 -1 or an average of 60% for captions and 94% for images) of constructs. Posts with 5 constructs in captions (average of 0.2% of posts) and images (average of 0% of posts) were extremely uncommon across influencer categories. The combination of 4 constructs in captions (average of 1% of posts) was slightly higher for captions but quite low in comparison to other combinations. While there are various theories regarding variable ordering in the HBM, a recent study regarding an influenza vaccination campaign found that variable ordering is complex but there is significant interaction between each variable [34]. Exposure to the vaccination campaign grounded in the HBM was positively associated with vaccine uptake behaviour [34]. A study by Guidry et al. (2019) that analyzed Instagram posts related to Zika virus found that messages contained relatively higher severity and susceptibility information but very few of the other constructs. Messages that only focused on the threat (severity and susceptibility) produced overall lower engagement, which may be due to the fear control response explained in the EPPM which results in maladaptive responses (Guidry et al., 2019). Thus, constructs in combination play an important role in message amplification and behaviour change regardless of the hierarchical order of the constructs. Our research found an alarmingly low number of post captions and images that contained 3–5 HBM constructs, which are all important aspects of influencing the uptake of public health measures and should be reflected in combination in messaging. The limited combination of HBM constructs among influencers, especially public health and government, would suggest lower acceptance and uptake of recommended behaviours and thus must be improved.

### Presence of threat and efficacy combined to impact the danger control process rarely used

Our research examined the combination of severity and cues to action/efficacy, susceptibility and cues to action/efficacy, and severity, susceptibility, and cues to action/efficacy and found posts rarely included these combinations. News media had the highest combination of susceptibility and cues to action/efficacy (3.6%) in captions with declining numbers across the other combinations and categories. The combination of threat (severity and susceptibility) and cues to action/efficacy in captions was highest for news media (1.2%) and between 0- 0.7% across the other influencer categories. Lower combinations were found within post images, with science communicators sharing images with higher combinations of threat and cues to action/efficacy (2%) with most combinations across influencers being 0 or under 1%. The EPPM theorizes the combination of threat and efficacy can elicit the danger control process, which impacts beliefs, attitudes, intentions, and behaviours in accordance with the message recommendations [36]. When the threat is not adequately communicated, even in the presence of efficacy information, motivation to act is low [36]. When messages contain high threat information but inadequate efficacy information, fear results and individuals tend to deny the threat and reject the message [36]. Finally, when no threat or efficacy information is present in messages, individuals do not consider the threat relevant and may not even be aware of the threat at all [36]. A recent study on COVID-19 news exposure found that as fear increased, protective behaviour decreased and weak efficacy messages were also associated with fear and defensive responses [67]. A Canadian COVID-19-related study found messages that targeted both threat and efficacy were associated with high intentions to follow government recommendations, adhering to physical distancing, and low fear [68]. The study authors recommended that crisis messages target both threat and efficacy to influence behaviour [68]. Our research across 33 influencers found posts rarely included combinations of threat and efficacy that would influence the danger control process. Crisis messages posted by Canadian influencers by large would influence the fear control process, which results in no threat perceived and thus no action or rejection of the message and recommended behaviour due to high fear without essential efficacy information [36, 38]. Individual behaviours, such as mask wearing, physical distancing, and vaccine uptake, are essential determinants of the burden of infectious diseases [69], and as such crisis messages must be designed to increase message acceptance and compliance with recommended behaviours.

### Practical implications

The findings of our research provide public health and other actors important information about providing crisis messages that reflect theory. Importantly, combining the constructs of the HBM and the EPPM to be able to adequately influence behaviour in crisis messages must be improved. Captions tended to include more constructs than images, representing an important area for improvement. Images are the most viewed aspect compared to captions and should convey the constructs to influence behaviour. Additionally, a focus specifically on combining threat and cues to action/efficacy to influence the danger control process where individuals understand the threat of the disease is high and understands how to protect themselves and feels able to do so. Finally, the response to messaging can be improved, especially with official actor messages. Public health had a lower than average engagement rate with their posts and overall the great proportion of comments were neutral, which was also seen for news media. Public health should consider monitoring social media to assess the effectiveness of messaging and incorporate aspects of messages shown to increase engagement and positive sentiment. Finally, partnering with brand influencers and local celebrities can help amplify public health messages within a captive and responsive audience.

### Limitations and future research

Using Instagram as the social media platform of choice was a source of limitations for this study. Namely, the manual data collection necessary when using Instagram limits the amount of data that can be collected when compared to automatically collecting data using a platform’s API. Additionally, young adults represent the largest group present on Instagram with older adults using the platform much less compared to Facebook. In addition, different audience segments may follow the various influencers included in this research, which may impact sentiment and engagement. As such, it is important to also evaluate crisis communication on other social media platforms for quality and public response, including engagement and sentiment. Additionally, the vast number of variables that can be collected related to Instagram posts, such as engagement metrics, comments and replies, and caption and image information provide a rich dataset that can be analyzed many ways. As such, our research presents descriptive statistics that describe the various variables included in this research. Future research should focus on further analyzing the relationships between variables to better understand if and how HBM constructs are related to engagement and sentiment. Finally, the HBM and EPPM include constructs that are centered on individual perception. To evaluate whether actors are using HBM and EPPM constructs in crisis communication, constructs were operationalized to reflect key features that can be identified in messages. Further research can involve participants to understand their perception related to various constructs and how they impact behavioural intentions related to COVID-19 and other public health emergencies.

## Conclusion

Theory-driven crisis communication plays an important role in mitigating the burden of disease during emerging infectious disease through impacting risk perception, efficacy, and ultimately the adoption of behaviours that reduce disease spread. Health Belief Model and Extended Parallel Processing mode constructs theorize that individuals will adopt behaviours to prevent disease when constructs are included in messaging. Our research found low use of constructs across crisis Instagram messages by a variety of influencer accounts during COVID-19. Moreover, extremely low combinations of constructs, including those that include threat and efficacy, were found. Neutral sentiment was highest for most accounts in response to crisis messages. Public health and government and news media were found to have lower than average engagement rates on their posts, while celebrities, brand influencers, and science communicators had higher engagement. These influencers represent important spokespersons with large followings and strong connections with their followers. Public health and government should partner with influencers who can amplify accurate public health information, influence risk perception, reach subpopulations, and increase trust. Overall, constructs should be combined in crisis messages to improve crisis communication, increase message acceptance, and influence risk perception and empower individuals to adopt risk protective measures.

## Abbreviations

API: Application Programming Interface
COVID-19: Coronavirus 2019
EPPM: Extended Parallel Processing Model
HBM: Health Belief Model

## Declarations

### Availability of data and materials

The datasets used and/or analysed during the current study are not publicly available due to additionally analysis being conducted but are available from the corresponding author on reasonable request.
