# Supplementary material for: Correction: A content analysis of Canadian influencer crisis messages on Instagram and the public’s response during COVID-19
Source: BMC Public Health. 2022 Oct 3;22:1848. doi: 10.1186/s12889-022-14203-8 (PMC9528870; doi:10.1186/s12889-022-14203-8)
Supplement: Supplementary file 1 — Additional file 1: Supplementary file 1. [file 12889_2022_14203_MOESM1_ESM.docx]

#### Results

#### Content Analysis

#### Health Belief Model Constructs in Captions and Images

The use of HBM constructs across influencer categories varied across post captions and images. In post captions (Table 5), severity was used least in posts by politicians (6%), public health and government (2%), science communicators (6%), and brand influencers and celebrities (2%). Cues to action/efficacy was used least in posts made by news media (9%). News media, politicians, and brand influencers and celebrities used barriers the most in their post captions (34%; 34%; 41% respectively). Cues to action were included most frequently in post captions by public health and government (33%) and science communicators (37%). Comparing the relationships between the use of HBM constructs in captions and influencer types was found to be statistically significant.

In post images (Table 6), severity was used least in posts made by politicians (9%), public health and government (2%), science communicators (7%), and brand influencers and celebrities (0%). Cues to action/efficacy was used least by news media (6%). News media used benefits (35%), politicians, science communicators, and brand influencers and celebrities used cues to action and efficacy (39%; 41%; 50% respectively), public health and government used barriers (39%) most frequently in post images. The relationship between HBM constructs in post images and influencer category was found to be statistically significant.

#### Combination of HBM Constructs Used in Captions and Images

When examining the number of HBM constructs used per post caption (Table 4.7), posts with no HBM constructs (n=0) and thus no combinations of constructs, were the most common for all influencer categories except public health and government and news media for which one was the most common number of HBM constructs in post captions.

**Discussion**

### HBM and EPPM Constructs Were Not Widely or Consistently Found in Instagram Captions or Images

Overall, the most frequent use of a construct was barrier-related information on Instagram captions made by news media (35%), politicians (34%) and brand influencers and celebrities (41%). Susceptibility information was not widely included with 17% or less of captions and 16% or less of images across influencer categories. Benefits, barriers, and cues to action/efficacy were incorporated inconsistently between influencer categories and captions and images. Past research examining influenza vaccine behaviour found that severity, susceptibility, barriers, and cues to action/efficacy together were significantly related to intention to get vaccinated (Guidry et al., 2020). Another study that examined COVID-19 vaccine behaviour found that self-efficacy was an important predictor of vaccine behaviours and play a mediating role with other constructs including barriers, benefits, and cues to action (Chen et al., 2021). Cues to action/efficacy were used in between 9-37% of captions and 6-50% of images, although 50% of images translates to seven instances of this construct in brand influencer images.

Table 5: Use of Health Belief Model Constructs Across Influencer Categories in Instagram Post Captions

| Influencer Category | Severity  n (%)^1^ | Susceptibility  n (%)^1^ | Benefits  n (%)^1^ | Barriers  n (%)^1^ | Cues to Action/Efficacy  n (%)^1^ | Total Constructs Used |
| --- | --- | --- | --- | --- | --- | --- |
| News Media | 179 (15%) | 208 (17%) | 301 (25%) | 414 (34%) | 103 (9%) | 1,205 |
| Politicians | 20 (6%) | 42 (13%) | 71 (21%) | 113 (34%) | 88 (26%) | 334 |
| Public Health and Government | 9 (2%) | 27 (6%) | 147 (30%) | 144 (30%) | 159 (33%) | 486 |
| Science Communicators | 31 (6%) | 62 (13%) | 97 (20%) | 121 (25%) | 182 (37%) | 493 |
| Brand Influencers and Celebrities | 1 (2%) | 2 (3%) | 20 (31%) | 26 (41%) | 15 (23%) | 64 |

^1^ % determined by dividing the use of each construct by the total number of uses of constructs across influencer category
*X*^2^= 334.166 on 16 degrees of freedom, p<0.05

Table 6: Use of Health Belief Model Constructs Across Influencer Categories in Instagram Post Images

| Influencer Category | Severity  n(%)^1^ | Susceptibility  n(%)^1^ | Benefits  n(%)^1^ | Barriers  n(%)^1^ | Cues to Action/Efficacy  n(%)^1^ | Total Constructs Used |
| --- | --- | --- | --- | --- | --- | --- |
| News Media | 85 (19%) | 56 (13%) | 155 (35%) | 127 (28%) | 25 (6%) | 448 |
| Politicians | 9 (7%) | 14 (11%) | 14 (11%) | 23 (18%) | 39 (30%) | 99 |
| Public Health and Government | 4 (5%) | 12 (14%) | 39 (46%) | 65 (77%) | 47 (28%) | 167 |
| Science Communicators | 27 (8%) | 58 (16%) | 56 (16%) | 71 (20%) | 149 (41%) | 361 |
| Brand Influencers and Celebrities | 0 (0%) | 1 (7%) | 4 (29%) | 2 (14%) | 7 (50%) | 14 |

^1^ % determined by dividing the use of each construct by the total number of uses of constructs across influencer category
*X*^2^= 221.794 on 16 degrees of freedom, p<0.05

Table 7: Use of Health Belief Model Constructs in Combination in Captions

| Number of Health Belief Model Constructs Per Post | News Media  n(%) | Politicians  n(%) | Public Health and Government  n(%) | Science Communicators  n(%) | Brand Influencers and Celebrities  n(%) | Average n(%) |
| --- | --- | --- | --- | --- | --- | --- |
| 0 | 320 (30%) | 248 (52%) | 133 (30%) | 270 (46%) | 40 (46%) | 1011 (38%) |
| 1 | 364 (35%) | 139 (29%) | 163 (37%) | 196 (33%) | 35 (40%) | 897 (34%) |
| 2 | 270 (26%) | 67 (14%) | 109 (25%) | 91 (15%) | 8 (9%) | 545 (21%) |
| 3 | 86 (8%) | 15 (3%) | 31 (7%) | 30 (5%) | 3 (4%) | 165 (6%) |
| 4 | 9 (0.9%) | 4 (1%) | 3 (1%) | 5 (0.8%) | 1 (1%) | 22 (0.8%) |
| 5 | 1 (0.1%) | 0 (0%) | 0 (0%) | 1 (0.2%) | 0 (0%) | 2 (0.01%) |
